# Round the Hospitals

**Published:** 1920-05-22

**Authors:** 


### ROUND THE HOSPITALS.

The King held the first open-air Investiture of the season in the Quadrangle of Buckingham Palace on May 12, and conferred decorations on the following members of the Nursing Services:—Royal Red Cross, First Class.—Miss Mary James, Q.A.I.M.N.S.R.; Misses Mary Bolderstone and Mary Pool, T.F.N.S. Royal Red Cross, Second Class.—Misses Dorothy Allen and Mabel Midrell, Q.A.I.M.N.S.R.; Misses Harriette Powell-Evans, May Slaney, and Annie Sly, Mrs. Ingram-

**ROUND THE HOSPITALS**—(*continued*).

Yelf, T.F.N.S.; Misses Annie Burr, Charlotte Johnson, Priscilla Roberts, and Frances Waugh, B.R.C.S.; Misses Sarah Daggor and Gertrude Spencer, Civil and War Hospital; Misses Clara Bartholomew, Helen Ellis, and Brenda Lea, the Viscountess Northcliffe, Misses Theresa Rice-Oxley, Sylvia Wadham-Stoll, Julia Swanston, and Amelia Watt, Mrs. Gertrude Woolley, V.A.D. All the above ladies, with Miss A. B. Smith, R.R.C., Matron-in-Chief, were subsequently received at Marlborough House by Queen Alexandra. Her Majesty was much better, but had not yet been allowed to drive out.

---

The King has awarded the Royal Red Cross to the following ladies in recognition of their valuable services in connection with the war, dated July 31, 1919. Royal Red Cross, First Class.—Miss G. L. Ball, A.R.R.C., Assistant Matron King George V. Hospital, Dublin; Miss B. M. Duff, A.R.R.C., Assistant Matron Military Hospital, Londonderry; Miss N. C. Stokes, Matron, Military Hospital, Tipperary. Royal Red Cross, Second Class.—Miss A. J. Boyd, Assistant Matron, Military Hospital, Londonderry; Miss S. E. Bradshaw, Officers' Hospital, Holywood; Miss E. Coun, Mercer's Hospital, Dublin; Miss P. Curtin, Matron, Mater Infirmorum Hospital, Belfast; Miss J. Drew, Assistant Matron, Sir Patrick Dun's Hospital, Dublin; Miss M. Dunne, Quiden Auxiliary Hospital; Miss S. F. H. Gilbert, Matron, Auxiliary Convalescent Hospital, Stillorgan; Miss M. H. Law, V.A.D.; Miss B. Leydon, Central Military Hospital, Cork; Miss H. M. Lowe Cart, Military Hospital, Curragh; Miss F. M. O'Driscoll, Special Military Hospital, Blackrock; Miss E. Scott, Central Military Hospital, Belfast; Miss A. Sproule, Sir Patrick Dun's Hospital, Dublin; Miss E. M. Studdert, Matron, Military Hospital, Fermoy.

---

The Queen visited the City on May 13, and received at the Mansion House the officials and members of the Queen Mary's Needlework Guild. A specially high daïs was erected in the Egyptian Hall, in order that all the numerous company might see the proceedings. Her Majesty, who was accompanied by Princess Mary and attended by Countess Fortescue and Mr. Harry Verney, was received by the Lord Mayor and Lady Mayoress, with the Sheriffs. Lady Harcourt expressed the desire of the Guild to enlist the energies of the new generation, and hoped the younger ladies would follow the example of Princess Mary. After speeches were over the Queen moved about among the company, recognised many who were present, and discussed plans for the future. Five ladies from the Leicester branch of the Guild were present, and were able to inform Her Majesty, to her great satisfaction, that a new subsidiary committee had been formed with the express purpose of working for the Queen's Maternity Home in Leicester, and would send it for the future one-tenth of all they collected. At the end of the proceedings the Queen had tea with the Lady Mayoress, and the general company were regaled in the ball-room.

---

Princess Marie Louise, Duchess of Argyle, who took the chair at the annual meeting of the University College Hospital Ladies' Association on May 12, gave very interesting testimony to the educative effect of taking part in such work. During the two years she has been in active connection with the Association she has come to an intense appreciation of those who devote their energies and lives to the alleviation of suffering. Her Royal Highness spoke stirring words on the duty of meeting with increased energy the present "crisis in the hospital life of the nation," and expressed her belief that the individual labours of the members of such associations were doing much to stave off that terrible and disastrous day when hospitals might have to be thrown upon the rates.

---

Prince Albert has accepted the office of President of the Queen's Hospital for Children, Bethnal Green, and presided last week at the annual meeting of the Governors. His Royal Highness previously made a careful survey of the institution, and took a great interest in what he described as "the excellent working arrangements of the staff." His cheering speech showed that he had not only studied the institution, but also the neighbourhood in which it is situated, and it was this which carried conviction when he spoke with confidence of the future of "one of the most important and most useful of the London hospitals." The need for voluntary help in the nursing department was disclosed in the statement made by Lord William Cecil that in consequence of the shortage in the supply of nurses they had been obliged to reduce the number of in-patients. We can think of no more powerful appeal to the leisured women in search of an interest in life than such an announcement.

---

News comes through of the first meeting of the General Nursing Council for Scotland, held on the 10th inst. at the office of the Scottish Board of Health, Edinburgh. The business was of a formal character. Sir Leslie Mackenzie, of the Board of Health, presided. Captain C. B. Balfour, of Newton Don, was appointed Chairman, and Miss Norah Milnes, B.Sc., Vice-Chairman. The interim Secretary is Mr. C. L. Farmer, of the Scottish Board of Health. The General Nursing Council for England and Wales held its first meeting the following day at the Ministry of Health.

---

The interests of nurses have not been overlooked by the Royal Sanitary Institute, who are organising the Health Congress to be held at Birmingham in July. Some time ago the College of Nursing was asked to appoint a delegate, and Miss Musson, R.R.C., Matron of the Birmingham General Hospital, was appointed to represent the College and to report on the proceedings. College members may therefore anticipate some useful and interesting information.

**ROUND THE HOSPITALS**—*(continued)*.

The nursing staff of the Great Northern Central Hospital is at present living in six dilapidated houses. These are both uncomfortable and inconvenient, and the Hospital Committee are doing all in their power to provide better quarters. The public and the theatrical profession joined hands with the Committee on Friday, May 14, and the result was an excellent entertainment at the Palladium and a well-filled house. We are left wondering what will be the next effort promoted by this valiant nursing staff in order to get an adequate roof over their heads. Their energy and determination are irresistible. Everyone on May 14, including a large number of sisters and nurses in boxes and stalls, appeared to be thoroughly enjoying the long and varied programme. Mr. Charles Gulliver had kindly lent the theatre and arranged the matinée, Sir George Elliott waived his contract rights, all the artistes gave their services, and Messrs. Fullers and Chocolat Lambert sent chocolates for sale. Miss Irene Vanbrugh contributed two delightful recitations, Manny and Roberts (the Messenger Boys from Broadway) and Miss Lily Long were all really amusing, and there were excellent dances and songs. Houdini, securely locked in a water-filled case, after a thrilling and endless minute emerged convincingly wet, and was welcomed with enthusiastic applause.

There is a good deal of unrest in New Zealand hospitals, and it is interesting to note that the lay papers are taking a very decided part in exposing the hardships under which nurses suffer. For instance, the *Christchurch Press* and the *Auckland Star* publish, with great exactitude, nurses' working hours at the Public Hospital, Auckland, and expose the fact that the change of shifts is so awkwardly arranged that nurses who work from 2 P.M. to 10 P.M. one day are put on to work from 6 A.M. to 3 P.M. the next. The lay papers are emphatic in condemning also what they call "the curious disregard of the nurses' comfort." In all these hospitals the eight-hour day or an approximation to it is in force, but it is evident enough that mechanical regulations will not protect nurses from defects in organisation. No experienced matron ever thought they would.

The new Hospitals Act in Tasmania will, it is feared, have the effect of closing most of the accouchement and nursing homes in the cities and country centres. The regulations, excellent in themselves, have been formed on purely theoretical lines, entirely disregarding present possibilities. The principals, who are for the most part nursing sisters, have been informed that no certificate will be granted them unless they can carry out certain structural alterations which, under existing building difficulties, are practically out of the question It is an object-lesson in the evil of precipitous legislation, and the Tasmanian deadlock may serve to explain the reluctance of English municipalities to undertake the regulation of nursing homes. Once again we are forced to the conclusion that legislation in regard to nursing matters demands very careful investigation in consultation with members of the nursing profession acquainted with the full bearing of all the facts, if it is not to do more harm than good.

The letter which we publish this week from the Secretary of the Manor House Orthopædic Hospital, Hampstead, exposes a difficulty which is rendering the work of carrying on special institutions extremely perplexing. The proper time in a nurse's training for her to take a special course of orthopædic work is after the completion of her general training. But the newly certificated general-trained nurse does not expect to step back into the status of a pupil. She expects, and rightly, a good salary. She is no help to a hospital which wants probationers for the performance of the routine work. On the other hand, probationers are getting very shy of binding themselves for two, or even one, years' service to an institution unable to grant them a full certificate. We are disposed to think that the Local Voluntary Aid Detachments ought to be able to help, and that if part-time reliefs could be organised, with a backbone of fully-trained and well-paid nurses, the difficulty might to some extent be got over.

Another institution where nursing salaries and conditions of service have recently been revised is the National Hospital for the Paralysed and Epileptic. The latest arrangements are that probationers are engaged for three years, two of which are to be spent at the National, and one at an affiliated general hospital. At the end of satisfactory service for this period a certificate of training in affiliated hospitals, recognised by the College of Nursing, will be issued. If probationers care to stay a further year at the National they will have free training in massage, Swedish remedial exercises, and medical electricity. Twenty pounds salary will be paid for the first year, and £24 for succeeding years, and a policy for an annuity of £10 at the age of fifty will be taken out with the Royal National Pension Fund for Nurses for each probationer, and handed to her with premiums paid to date at the end of three years. Staff nurses at the National receive £50 per annum, or £24 if given free training in massage, S. R. E., and electricity. Ward sisters' salaries are £60, rising by £5 increments to £80. The night sister, home sister, electrical sister, housekeeping sister, etc., have all had their salaries revised recently.

The Children's Jewel Fund is a movement which has much good work to its credit. The Marchioness of Tweeddale presided, in the absence of Princess Christian, over a meeting for the inauguration of a new branch, intended to promote maternity and child welfare throughout the Highlands by the provision of trained nurses. The Hon. Sir Arthur Stanley mentioned that at the recent meeting of the International League of the Red Cross Societies at Geneva it had been decided that the best way to carry on their work lay in preventing the present appalling infant mortality in all countries, and a

**ROUND THE HOSPITALS**—*(continued)*.

particular town in the Highlands had been spoken of as the place where this kind of work was most needed. He would not name it, but it was *not* Inverness. The special difficulties to be encountered are deficiency of doctors and lack of transport, but nurses have, as we all know, an almost miraculous power of getting by hook or by crook to their cases, and the right course is to send well-trained, well-paid nurses to organise aid for the mothers. If the Children's Jewel Fund can accomplish this it will do well indeed.

---

The Hertfordshire County Nursing Association suggests, with regard to the midwifery grant made by the County Council, that financial assistance based upon deficits does not encourage local associations to become financially self-supporting and desires that the distribution of this grant should be placed within the discretion of the Association, and that for this purpose a total sum should be paid to it early in the year, to be applied solely towards the cost of providing midwives where necessary, the unexpended part of such sum in any year to be repaid to the County Council. The Association suggests a sum of £400, and the Health Committee of the County Council expresses acquiescence with the proposal.

---

If any there be who deem the present cult of motherhood overdone and superfluous, let them study the vital statistics, and realise that the process of making childbirth less arduous is already bearing rich fruit. In the last quarter of last year the birth-rate for England and Wales was only 15.9 per 1,000. Last week the returns from ninety-six large towns revealed that the rate had risen to 28.8 per 1,000. There is still much to be done, for the infant mortality rate, however shrunk, is highly discreditable. But the process of extending aid and encouragement to mothers evidenced in the new hostels for lying-in mothers springing up all over the country, and in the efforts of countless voluntary agencies, has already lifted some of the burdens from the shoulders of married folk, and will, we trust, have still more effect in the future.

---

Few hospitals have a more admirably organised Linen Guild than Swansea. Lady Dynevor took the chair at the annual meeting on May 5, and the Duchess of Beaufort, with a large gathering of supporters, was present to hear the tale of the Guild's successes. It has now forty branches, and no fewer than 3,508 members. During the eight years of its existence it has benefited the Swansea Hospital to the extent of £10,000, and now provides for over 300 beds. The hospital, which serves a wide district in West Wales, has a waiting-list of 400 cases, but the Parc Wern Hospital extension will provide an additional 100 beds, which will, of course, add to the labours of the Guild. The Linen Guilds do much more than provide useful articles. They point the way to the extension of voluntary hospital work by calling upon each subscriber not only to give, but to do.

---

History has continually to be re-written in accordance with the advance of science. The latest addition to our stock of information about British sovereigns is the American discovery that Queen Elizabeth was a life-long invalid, never sound in body or nerves. Mr. Frederic Chamberlain went to the pains of preparing a statement of all that could be discovered about the various illnesses of the Queen, and submitted it to several leading medical authorities for their opinion. This medical record was read by the author at the Royal Institution on May 1. It shows that Elizabeth was continuously ill from her fourteenth year till she came to the throne ten years later, and that, in fact, she never altogether recovered. She was a victim of small-pox, which ruined her looks, and was subject to fits in which she frequently remained unconscious for hours.

---

The Norwood Cottage Hospital confers a great boon on the residents in that locality, for out of the 532 patients treated during last year more than half belong to Upper Norwood. Yet at the annual meeting Mr. W. F. Turner, the President, stated that a great many people in the neighbourhood are unaware of its existence. The managers project a house-to-house visitation undertaken by ladies to bring its needs to the notice of the public within that area, and trust to this to make up the extra expenditure entailed by increased prices. The average total cost of each in-patient per week has risen from £1 14s. 3¾d. in 1918 to £2 11s. There must be more than 3,000 inhabitants in Norwood.

---

At the last meeting of the committee of the Whitehaven and West Cumberland Infirmary a letter was received from Miss Lily Evans, the matron of the infirmary, stating that, in consequence of impaired health, she felt it her duty to resign her appointment, and asking to be released from duty in a month. Her resignation was accepted with regret. Miss Evans has held the appointment for about fifteen years. During that period she has taken a leading part in modernising the infirmary, and she leaves it in a satisfactory state of efficiency.

---

The town of Mons was the scene of a picturesque ceremony lately, when the Hon. Angela Manners, A.R.R.C., presented, on behalf of the British Government, "Certificates of Gratitude," signed by Queen Alexandra, from the British Red Cross and St. John Ambulance Association to certain Belgian ladies who after the battle of Mons gave their houses for hospitals and acted as nurses to the English wounded. The British Military Attaché was present, with the municipal authorities of Mons, the Governor of the Province, and other notables. The "Manners Ambulance," established by the Hon. Angela Manners early in the war, was warmly eulogised by more than one speaker.

